# Studies on the Electrochemical Behavior of Thiazolidine and Its Applications Using a Flow–Through Chronoamperometric Sensor Based on a Gold Electrode

**DOI:** 10.3390/molecules16097608

**Published:** 2011-09-06

**Authors:** Lai-Hao Wang, Wen-Jie Li

**Affiliations:** Department of Medical Chemistry, Chia Nan University of Pharmacy and Science, 60 Erh-Jen Road, Section 1, Jen Te, Tainan 71743, Taiwan

**Keywords:** electrochemical behavior, thiazolidine, chronoamperometric sensor, gold electrode

## Abstract

The electrochemical behaviors of thiazolidine (tetrahydrothiazole) on gold and platinum electrodes were investigated in a Britton-Robinson buffer (pH 2.77–11.61), acetate buffer (pH 4.31), phosphate buffer solutions (pH 2.11 and 6.38) and methanol or acetonitrile containing various supporting electrolytes. Detection was based on a gold wire electrochemical signal obtained with a supporting electrolyte containing 20% methanol-1.0 mM of phosphate buffer (pH 6.87, potassium dihydrogen phosphate and dipotassium hydrogen phosphate) as the mobile phase. Comparison with results obtained with a commercial amperometric detector shows good agreement. Using the chronoamperometric sensor with the current at a constant potential, and measurements with suitable experimental parameters, a linear concentration from 0.05 to 16 mg L^−1^ was found. The limit of quantification (LOQ) of the method for thiazolidine was found to be 1 ng.

## 1. Introduction

Thiazolidine (tetrahydrothiazole)-4-carboxylic acid derivatives were evaluated for their ability to inhibit neuraminidase (NA) of influenza A virus and human prostate cancer cell lines [1,2]. Some 4- thiazolidine derivatives have been tested for their antimicrobial, anti-inflammatory and antioxidant activities, and evaluated for the potency of their inhibitory effect on the production of nitric oxide (NO) [3,4,5,6,7,8]. Many studies [9,10,11,12,13,14,15,16,17,18] report the synthesis of thiazolidine derivatives as anti-cancer pharmaceuticals, however, almost all these studies focus on synthesis methods, chemical composition, and their biological activities, but there is scant chromatography and electrochemical literature reporting thiazolidine determinations [19,20,21].

Thiazolidine PG-15 was studied in rat plasma by high-performance liquid chromatography with UV detection at 385 nm method and its limit of quantification (LOQ) was 62.5 ng mL^−1^ [19]. A gas chromatography-electron impact ionization mass spectrometry (GC-EI-MS) method involved derivatization of thiazolidine-4-carboxylic acid with alcohol/chloroformate to produce 4-carboxylate derivatives [20]. The voltammetric adsorption and desorption of other thiols such as cysteine has been investigated at a gold electrode, since cysteine contains a sulphydryl (–SH) group [22,23]. Because thiazolidine is typically considered a starting material, no analytical method has been previously reported in literature monitoring its pharmaceutical levels. In this context, the aim of this work was to develop a specific and sensitive electrochemical analytical method to quantify thiazolidine. Metals like platinum and gold, owing to their high purity, easy machinability, and simple fabrication in a variety of geometric configurations (wires, rods, flat sheets, and gauzes) can be prepared as working electrodes of suitable size for use in flow cells. Furthermore, there were no reports in the literature concerning the determination of thiazolidine. Our investigation thus involved three approaches: (1) comparison of the sensitivity of gold and platinum electrodes for determination of thiazolidine; (2) cyclic voltammetry and differential pulse voltammetry were used to elucidate the electrochemical behavior of thiazolidine at a gold microelectrode; (3) design of electrochemical flow cell devices which were used for studying the flow of thiazolidine through gold electrode electrochemical processes; a chronoamperometric flow cell was used to characterize the sensor for analytical applications, regarding the quantitative determination of thiazolidine. 

## 2. Results and Discussion

### 2.1. Electrochemical Behavior of Thiazolidine at an Au Electrode

**Scheme 1:**
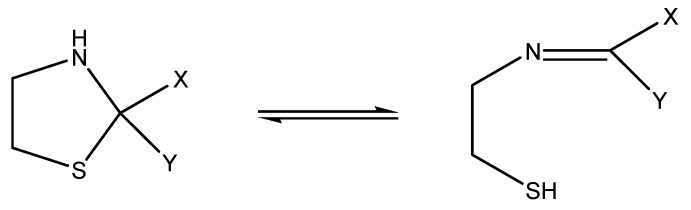
Tautomeric equilibrium.

Thiazolidine is normally in tautomeric equilibrium with an acyclic thiol form (Scheme 1) [24]. Thiols are easily oxidized anodically to disulfides [25]. In order to arrive at the optimum conditions for thiazolidine determination, there are several factors such as pH, supporting electrolytes, and working electrode which should be considered.

Comparative tests of suitable solvents, supporting electrolytes and pH-levels such as Britton-Robinson buffer (pH 2.77–11.61), phosphate buffer (pH 2.11 and 6.38), acetate buffer (pH 4.31), and methanol or acetonitrile containing various supporting electrolytes were conducted (Table 1). From Table 1 it can be seen that thiazolidine could not be determined in nonaqueous solvents (100% methanol or acetonitrile) on either Au or Pt electrodes due to the higher background in nonaqueous solvents than in aqueous-organic ones. The peak current for oxidation of thiazolidine in LiClO_4_/30% MeOH (122.1 μA) was higher than that in LiClO_4_/30% CH_3_CN (85.31 μA). In order to compare the electroanalytical utility of Au and Pt electrodes, we analyzed thiazolidine using cyclic voltammetry (CV).

**Figure 1:**
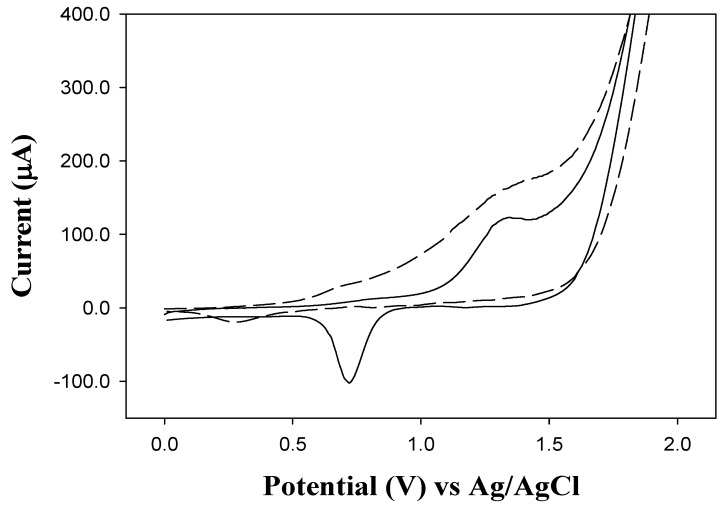
Cyclic voltammograms of thiazolidine (9.09 × 10^−4^ M) in aqueous protic solvent (methanol) containing lithium perchlorate (LiClO_4_/30% MeOH), scan rate, 100 mV/s. Solid line (−) is the Au electrode, medium dashed line (- -) is the Pt electrode.

**Figure 2:**
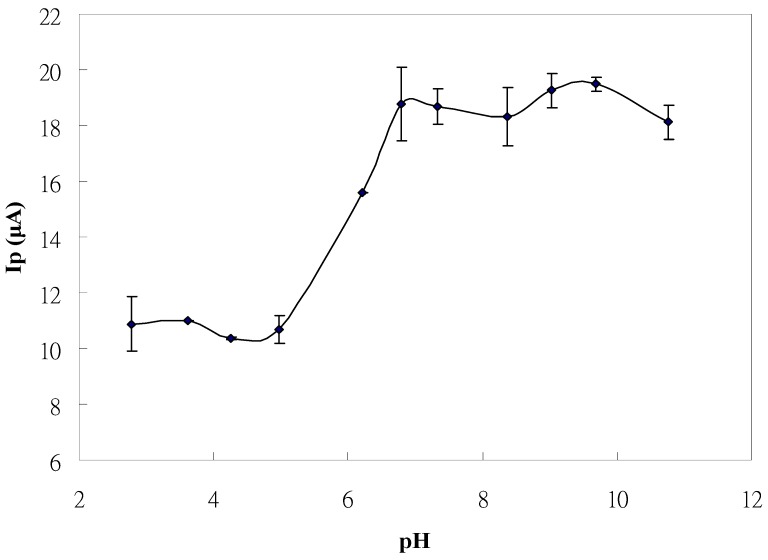
The effect of pH on the response current of 1.58 × 10^−4^ M thiazolidine in Britton-Robinson buffer at a gold electrode; differential pulse voltammetry; scan rate 10 mV/s.

**Figure 3:**
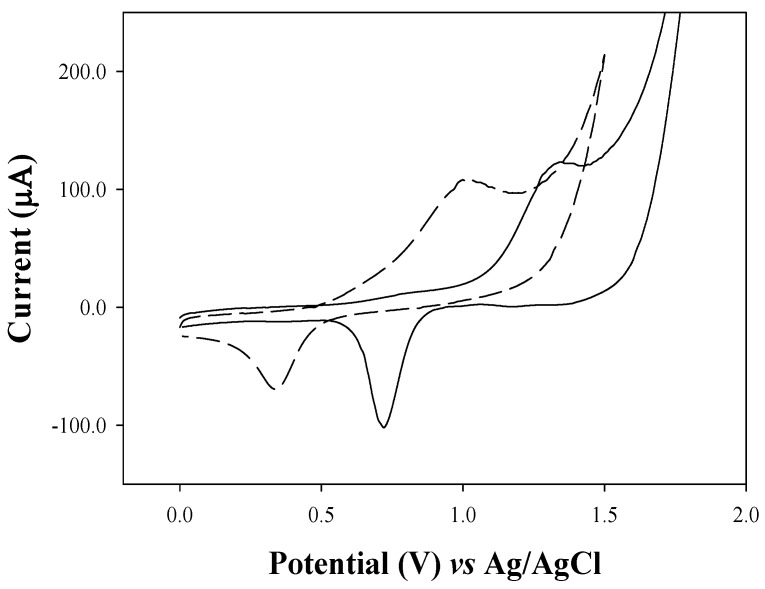
Cyclic voltammograms of thiazolidine (9.09 × 10^−4^ M) on Au electrode in different supporting electrolytes, scan rate, 100 mV/s. Solid line (−) is LiClO_4_/30% MeOH, medium dashed line (- -) is Britton-Robinson buffer (pH 6.94).

Figure 1 shows that the Au electrode gave a better-defined oxidation peak than that of the Pt electrode, therefore, we chose the Au electrode to determine thiazolidine. Differential pulse voltammograms of thiazolidine in Britton-Robinson buffered solution in the pH range of 2.77–11.61 at Au electrode show one well-defined oxidation. The plot of Ip *vs.* pH (Figure 2) and the maximum peak current were obtained between pH 6.80 and 10.76. For analytical purposes, the best supporting electrolyte for the determination of thiazolidine is Britton-Robinson buffer pH 7-8. The peak potential of thiazolidine in Britton-Robinson buffer solution (pH 6.94) + 0.9995 V is less positive than in LiClO_4_/30% MeOH + 1.341 V (Figure 3). 

### 2.2. Cyclic Voltammetry (CV)

**Figure 4:**
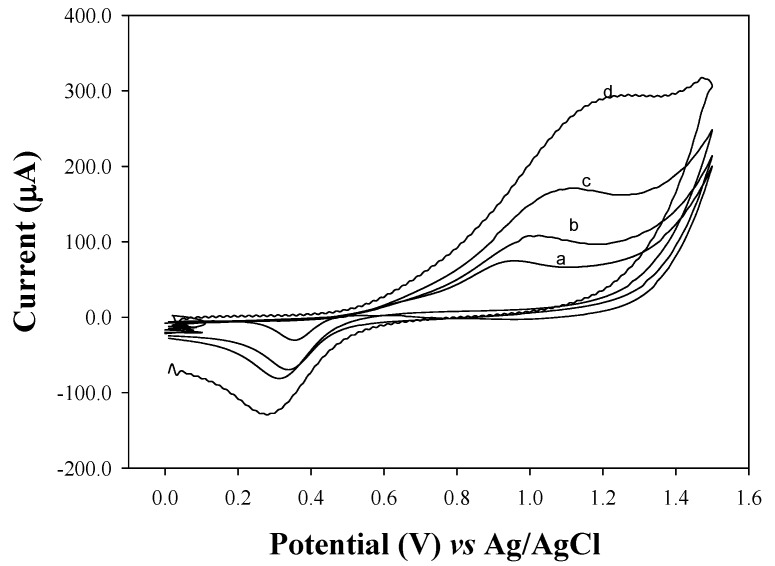
Cyclic voltammograms of thiazolidine (9.09 × 10^−4^ M) on Au electrode in Britton-Robinson buffer (pH 6.94) at different scan rates (a) 50 mV/s; (b) 100 mV/s; (c) 200 mV/s; (d) 400 mV/s.

Cyclic voltammograms were recorded at different scan rates as shown in Figure 4. The effect of scan rate on the electrooxidation of thiazolidine was examined in aqueous-methanol in the range of 10 mV/s to 800 mV/s. 

**Figure 5:**
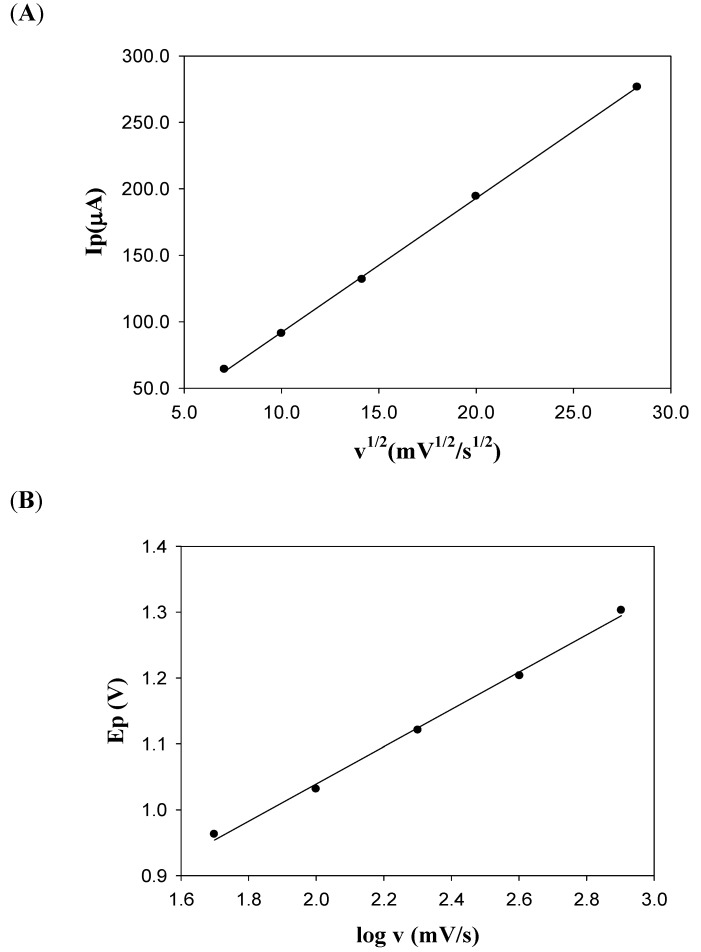
(**A**) Magnitude of the peak current, Ip, for thiazolidine (9.09 × 10^−4^ M) oxidation as a function of square root of scan rate (v ^1/2^) and (**B**) peak potentials of thiazolidine oxidation as a function of logarithm of scan rates (v).

In those cases the oxidative peak current was proportional to the square root of the scan rate. From Figure 5(A), good linearity was observed between the peak height (current) and the square root of scan rate; the regression equation being *y* = 10.1 *x −* 8.72; the correlation coefficient r = 0.9982). Under these conditions the oxidative peak currents were diffusion controlled. The relationship between peak potential and logarithm of scan rate [Figure 5(B), *y* = 0.283 *x* + 0.472; the correlation coefficient r = 0.9998] can be used to estimate roughly the number of electrons involved in the catalytic oxidation.

### 2.3. Optimum Conditions for Liquid Chromatography

**Figure 6:**
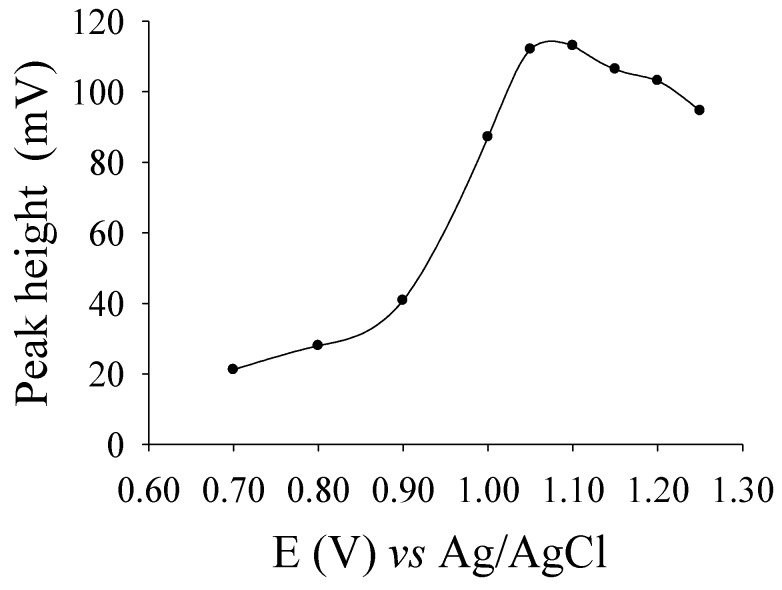
Hydrodynamic voltammograms obtained for thiazolidine (1 mg/L) flow through a flow cell system on Au electrode and methanol-water (20:80, *v/v*) containing 1 mM phosphate buffer (pH 6.87) as eluent.

Various ratios of methanol-water containing 1 mM phosphate buffer (pH 2.11–6.87) were prepared. After various studies of the retention behavior of the thiazolidine, baseline separation was achieved. Methanol-water (20:80,V/V) containing phosphate buffer (pH 6.87) was found to be the best eluent for a good sensitivity, higher than that observed with other eluents, therefore phosphate buffered solution was chosen for the determination of thiazolidine. In order to determine the optimum applied potential for electrochemical detection, following HPLC, hydrodynamic voltammograms were constructed for thiazolidine (Figure 6). The maximum current, measured as peak height, was achieved about at a potential between + 1.0 V and 1.1 V). The voltammetric detector was operated at + 1.1 V. Using the injection valve, 20 μL of the prepared standard solutions were chromatographed under the operating conditions described above. 

### 2.4. Sensitivity of Amperometric Detector and UV Detector for Liquid Chromatography

**Figure 7:**
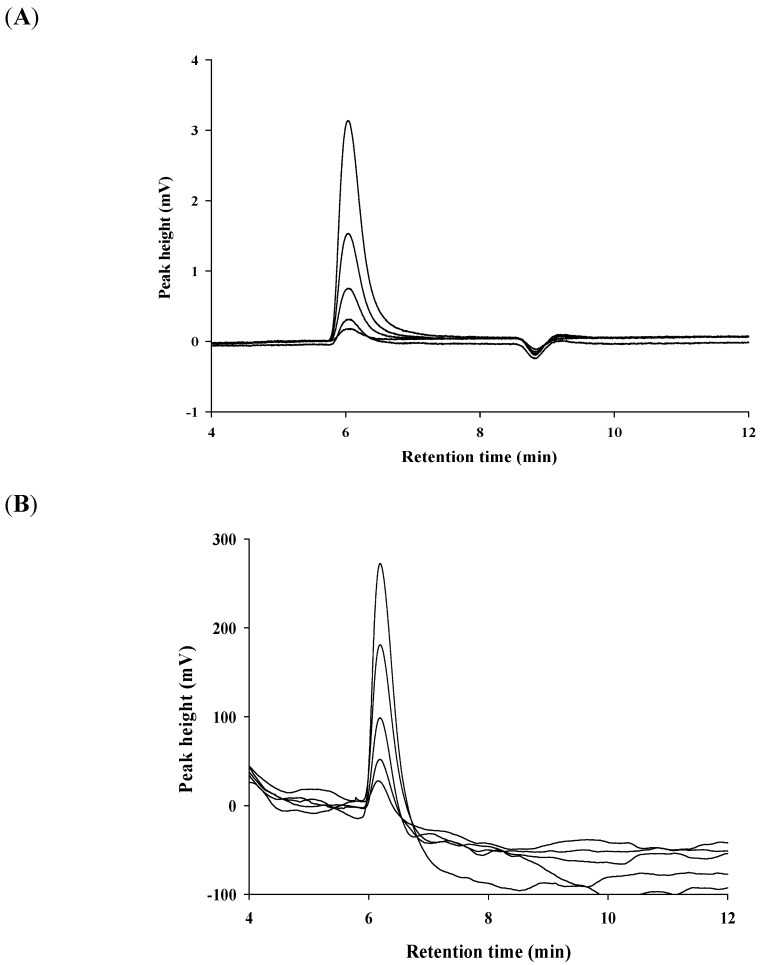
Chromatograms obtained to produce calibration graph for thiazolidine (**A**) LC-UV, (**B**) LC-ECD.

The calibration plots obtained by plotting the peak area against the concentration of thiazolidine showed good linearity over the range 0.05 to 6.40 μg mL^−1^. The regression equation was y =11.59 × −2.041 (correlation coefficient r = 0.9999) and the range 0.20–6.40 μg mL^−1^ for LC-UV; y =1120 × +355867 (correlation coefficient r = 0.9953) for LC-ECD. Our proposed LC-ECD method was used to determine thiazolidine. The limit of quantification (LOQ) of the method was found to be 1 ng for thiazolidine. Chromatograms comparing LC-UV and LC-ECD for thiazolidine show that the sensitivity for the thiazolidine investigated was about two orders of magnitude higher with LC-ECD than with LC-UV detection [Figure 7(A) and (B)]. 

### 2.5. Application to Thiazolidine Using a Flow—through Chronoamperometric Sensor

**Figure 8:**
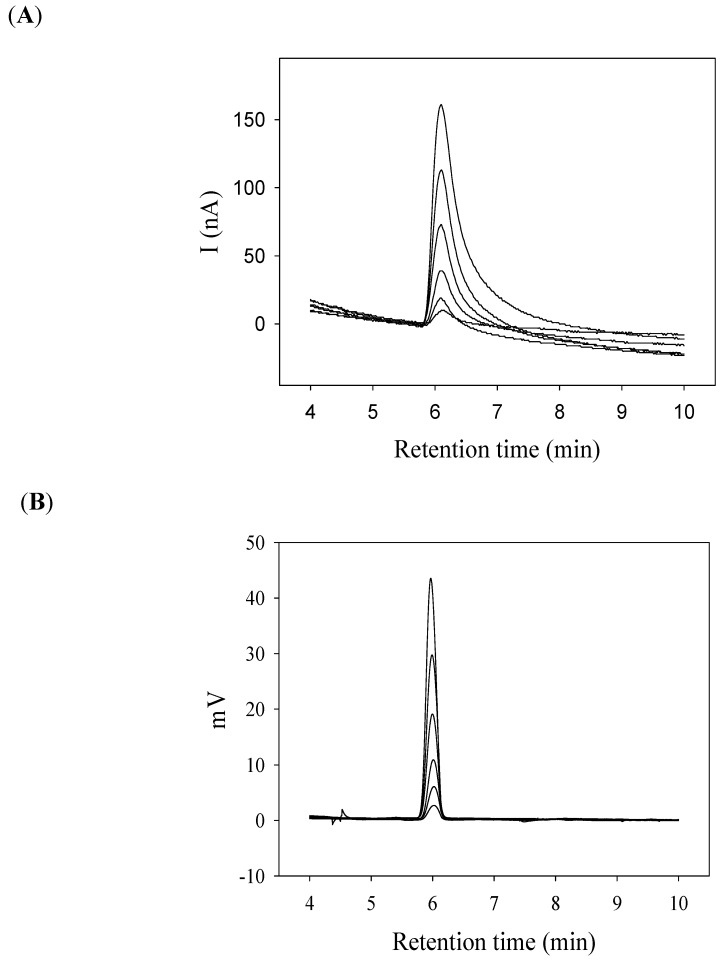
Comparison of (**A**) chronoamperometry, current *vs.* time (retention time 6.10 min) (**B**) chromatogram (retention time 6.02 min); using flow cell system on Au electrode and methanol-water (20:80, *v/v*) containing 1 mM phosphate buffer (pH 6.87) as eluent.

Chronoamperometry is a potentiostatic method for the measurement of the current that flows through the working electrode, measured as a function of time [Figure 8(A)]. The electrical signal over time was transferred and calculated by means of a SISC chromatogram data integrator and thus a chromatogram was obtained [Figure 8(B)]. From Figure 8(A) and (B), it can be seen that the chromatogram (retention time 6.02 min) and graphic chronoamperometry (current-time 6.10 min) were close to each other in time. The calibration curve method executes the quantification of an unknown sample by acquiring a regression equation which represents the relationship between the peak intensity (peak height) in current mode of target analyte and concentration from graphic chronoamperometry of thiazolidine standards whose concentration are already known. 

The calibration plots obtained by plotting the peak area against the concentration of thiazolidine showed good linearity over the range 0.05 to 6.40 μg mL^−1^. The regression equations were y = 51784 *×* +82951 (correlation coefficient, r = 0.9961); the range 0.50–16 μg mL^−1^ and y = 53342 × +65153 (correlation coefficient, r = 0.9945) for Figure 8(A) (chronoamperometry) and Figure 8(B) (chromatogram), respectively. Hence in this study, determination of the concentration of thiazolidine was accomplished by means of flow cell chronoamperometric sensor procedure.

## 3. Experimental

### 3.1. Materials and Apparatus 

The sample of thiazolidine was purchased from Acros Organics (Geel, Belgium). Supporting electrolytes were obtained as follows: tetrabutylammonium tetrafluoroborate (Bu_4_NBF_4_) and lithium perchlorate (LiClO_4_) ‒ Acros Organics, Thermo Fisher Scientific (Geel, Belgium); tetraethyl- ammonium tetrafluoroborate (Et_4_NBF_4_) ‒ E. Merck Chemical Co., (Darmstadt, Germany); tetrabutylammonium perchlorate (Bu_4_NClO_4_) and tetraethylammonium perchlorate (Et_4_NClO_4_) ‒ Tokyo Chemical Industry Co., Ltd (Tokyo, Japan). Voltammetric measurements were performed using an electrochemical trace analyzer (Model 394; EG&G Princeton Applied Research, Princeton, NJ). A high-performance liquid chromatography (HPLC) system (LC-10 AD_vp_; Shimadzu, Kyoto, Japan) containing a Rheodyne 7125 injection valve with a 20-μL sample loop coupled to an amperometric detector (Decade II; Antec (Leyden) B.V., Zoeterwoude, The Netherlands) and a spectrophotometric detector (L-7420; Hitachi, Japan) was used. The chronoamperometric sensor system used for the experiments was home-made and consisted of a potentiostat/galvanostat (263A; EG&G Princeton Applied Research, Princeton, NJ, USA) controlled potentiostat, coupled to a spectrophotometric detector (L-7420; Hitachi, Japan). The flow cell was designed with the following electrodes: an Ag/AgCl/0.1 M KCl reference electrode, a stainless steel auxiliary electrode, and a gold electrode (length 8 cm, i.d. 0.3 mm) as working electrode for detecting thiazolidine. The flow-through electrolysis cell was prepared in our laboratory as previously described [22] to detect thiazolidine. All solvents and analytes were filtered through 0.45-μm cellulose acetate and polyvinylidene fluoride syringe membrane filters, respectively. Chromatograms of thiazolidine were registered and peak height was calculated using a chromatogram data integrator (Scientific Information Service Corp., Davis, CA, USA). All other reagents were locally purchased and of analytical grade.

### 3.2. Procedures

#### 3.2.1. Voltammetric Measurements

The two voltammetric techniques, differential pulse voltammetry and cyclic voltammetry, were all performed on a gold electrode. Voltammograms of thiazolidine were taken on a gold electrode in lithium perchlorate (pH 6.01), acetate buffer (pH 4.31), phosphate buffer solutions (pH 2.11 and 6.38) and Britton and Robinson buffer solutions (pH 2.77–11.61). Cyclic voltammetry and chronoamperometry were carried out on an Au electrode in order to monitor potential *vs*. current of the electrode concurrently with the electrochemical measurements.

#### 3.2.2. Determining Thiazolidine Using a Flow-through Chonoamperometric Detector

The amperometroc detector was operated at 0.7–1.3 V for hydrodynamic voltammograms. By means of the injection value, 20 μL of the prepared standard solution was chromatographed under the operating conditions described above. Quantitation was based on the peak area of the sample.

#### 3.2.3. Determining Thiazolidine Using Liquid Chromatography with UV Detector

Stock solution of standard was prepared by dissolving thiazolidine (10 mg) in methanol (10 mL). Working standard solutions were prepared from a stock standard solution in methanol in the range 0.05–6.4 μg mL^−1^. RP- HPLC was performed on a Phenomenex Luna C_18_ (5 μ, 250 × 4.6 mm) column eluted with methanol - water (20:80, V/V, containing 1.0 mM phosphate buffer, pH 6.68) as the mobile phase at flow rate of 1 mL/ min. Detection after separation on the Phenomenex Luna C_18_ column was carried out using an ultraviolet detector set at 216 nm. 

## 4. Conclusions

We have constructed a gold electrode for use as a flow-through chonoamperometric sensor for the determination of thiazolidine. The sensor affords current, potential and direct determination of thiazolidine at the gold electrode. When thiazolidine was determined with the method proposed the results obtained were comparable with those obtained with a commercial amperometric detector, thus the proposed analytical method offers a valid and economical alternative to UV detection of thiazolidine. 

## Figures and Tables

**Table 1:** Effect of supporting electrolytes on the cyclic voltammetric peak potential and peak current of thiazolidine at gold and platinum electrodes.

Supporting electrolytes	Thiazolidine			
	Au		Pt	
	Potential (V)	Current (*μ*A)	Potential (V)	Current (*μ*A)
Bu_4_NBF_4_/100% CH_3_CN	ND	ND	ND	ND
Et_4_NBF_4_/100% CH_3_CN	ND	ND	ND	ND
Bu_4_NClO_4_/100% CH_3_CN	ND	ND	ND	ND
Et_4_NClO_4_/100% CH_3_CN	ND	ND	ND	ND
LiClO_4_/100% CH_3_CN	ND	ND	ND	ND
LiClO_4_/100% MeOH	ND	ND	ND	ND
Et_4_NBF_4_/30% CH_3_CN	1.344	162.7	ND	ND
Et_4_NClO_4_/30% CH_3_CN	1.336	86.69	ND	ND
LiClO_4_/30% CH_3_CN	1.308	85.31	ND	ND
LiClO_4_/30% MeOH	1.341	122.1	1.332	155.9

ND: Not determined. Supporting electrolytes: tetrabutylammonium tetrafluoroborate (Bu_4_NBF_4_); tetraethylammonium tetraflorborate (Et_4_NBF_4_); tetrabutylammonium perchlorate (Bu_4_NClO_4_); tetraethylammonium perchlorate (Et_4_NClO_4_); lithium perchlorate (LiClO_4_).
